# Carcinoma In Situ Skip Lesions of the Uterine Corpus in Cervical Squamous Cell Carcinoma: Impact on Target Setting in Radiotherapy

**DOI:** 10.1002/cnr2.70751

**Published:** 2026-11-05

**Authors:** Mayumi Harada, Yasuhito Hagiwara, Takanobu Kabasawa, Hiraku Sato, Mayumi Ichikawa, Hiroko Akamatsu, Takashi Kaneko, Natsuko Yano, Ken Uematsu, Masanori Komatsu, Taiyo Tanae, Kenji Nemoto, Masashi Koto

**Affiliations:** ^1^ Department of Radiation Oncology Yamagata University Faculty of Medicine Yamagata Yamagata Japan; ^2^ Department of Pathology Yamagata University Faculty of Medicine Yamagata Yamagata Japan

**Keywords:** radiotherapy, uterine cervical neoplasms, uterus

## Abstract

**Background:**

In definitive radiotherapy for FIGO Stage I–III cervical cancer, external beam radiotherapy and/or brachytherapy are used—alone or in combination with concurrent chemotherapy depending on stage—with target delineation largely guided by clinically apparent disease. However, discontinuous carcinoma in situ (CIS) “skip lesions” within the uterine corpus are well described in cervical adenocarcinoma, whereas their presence in cervical squamous cell carcinoma (SCC) has not been systematically evaluated.

**Aims:**

This study aimed to investigate the presence or absence of CIS skip lesions in the uterine corpus of patients with cervical SCC.

**Methods and Results:**

This retrospective study included all patients who underwent total or radical hysterectomy for cervical SCC at Yamagata University Hospital between January 2013 and April 2020. Patients who underwent prior radiotherapy or preoperative chemotherapy, or had non‐squamous histological components, were excluded. Fifty patients were enrolled. Pathological examination of serially sectioned uterine specimens was conducted to identify skip lesions, defined as discontinuous CIS lesions in the uterine corpus, as confirmed by a pathologist and a radiation oncologist. Pathological evaluation showed that 29 patients (58%) had FIGO Stage I disease, 6 patients (12%) had FIGO Stage II disease, and 15 patients (30%) were classified as FIGO Stage IIIC1. Preoperative imaging studies did not identify any skip lesions in the uterine corpus. However, one patient (2%; Clopper–Pearson 95% confidence intervals: 0.05%–10.5%) presented with a histologically confirmed CIS skip lesion located in the uterine corpus, approximately 2 cm cranial to the primary tumor margin, that was undetectable on imaging. The primary tumor after biopsy measured 3 mm and was diagnosed as non‐keratinizing SCC without > 1/3 stromal invasion. Lymphovascular space invasion, parametrial invasion, and corpus invasion were not observed.

**Conclusion:**

Although rare, CIS skip lesions in the uterine corpus can occur in patients with cervical SCC.

## 1 Introduction

Radiotherapy plays a major role in the curative treatment of cervical cancer. According to the International Federation of Gynecology and Obstetrics (FIGO) classification [1], treatment strategies are determined by disease stage. For early‐stage disease (FIGO IA1‐IB1), radiotherapy—either brachytherapy alone or external beam radiotherapy (EBRT) combined with brachytherapy—may be used as definitive treatment [2]. For locally advanced cervical cancer, radical radiotherapy consisting of EBRT with concurrent chemotherapy followed by brachytherapy is recommended [3]. In metastatic disease, palliative radiotherapy is administered in addition to systemic therapy [4].

EBRT typically involves whole‐pelvic irradiation, including the uterus, whereas intracavitary brachytherapy has conventionally targeted the entire uterus using point A‐based dose prescription. However, in recent years, brachytherapy dose prescription has increasingly shifted toward the high‐risk clinical target volume (HR‐CTV). This volume encompasses the tumor and the entire cervix contoured on CT images for brachytherapy planning, and is defined using MRI findings, pelvic and rectal examinations, and other clinical assessments [5].

HR‐CTV‐based dose prescription enables more accurate coverage of tumor regions that may be insufficiently irradiated with conventional point A‐based methods. Furthermore, when point A lies outside the tumor volume, unnecessary high‐dose exposure can be avoided. This individualized approach is expected to improve local tumor control while minimizing toxicity to surrounding organs [3]. Nevertheless, strict adherence to HR‐CTV‐based prescription may exclude a substantial portion of the uterine corpus from the target volume, potentially resulting in underirradiation of the corpus. Consequently, microscopic lesions below the resolution limits of CT or MRI may remain untreated, posing a risk of local recurrence. During whole‐pelvic irradiation, the increasing use of intensity‐modulated radiotherapy (IMRT) has enabled highly conformal dose distributions. To reduce doses to organs at risk, the uterine corpus—when no tumor is visible on imaging—is sometimes excluded from the target volume [6, 7, 8].

Previous reports have shown that 45% of patients with cervical adenocarcinoma who underwent conization for carcinoma in situ (CIS) with positive margins had CIS skip lesions in the uterine corpus [9], underscoring the need to consider irradiation of the corpus. However, the incidence of CIS skip lesions in the uterine corpus among patients with cervical squamous cell carcinoma (SCC) remains largely unknown, and comprehensive investigations are lacking [10].

Therefore, the aim of this study was to comprehensively evaluate the excised uteri of patients who underwent total hysterectomy for cervical SCC and determine the frequency of CIS skip lesions in the uterine corpus.

## 2 Methods

### 2.1 Ethics Statement

This study was conducted in accordance with the Declaration of Helsinki and was approved by the Ethics Committee of our hospital (Approval No. 2019‐364). Because of the retrospective nature of the study, informed consent was waived, although an opt‐out option was provided on the facility's website.

### 2.2 Patient Selection

Fifty patients who underwent total or radical hysterectomy for cervical SCC at Yamagata University Hospital between January 2013 and April 2020 were included in this study, provided that the corresponding pathological specimens were available. Patients with a history of prior pelvic radiotherapy or those who had received preoperative chemotherapy were excluded because these conditions could complicate the pathological assessment. In addition, cases with non‐squamous histological components were excluded to minimize the risk of overestimating the frequency of CIS skip lesions. Tumor staging was performed in accordance with the FIGO classification, 2018 Edition.

### 2.3 Imaging and Pathological Evaluation

During surgical specimen processing, the extent of uterine tumor spread observed on gross pathology was compared with the findings on preoperative CT and MRI using cutout diagrams to confirm concordance at the centimeter scale. Serial transverse sections of the uterine corpus were used to determine the presence or absence of skip lesions. The uterine corpus was serially sectioned at 5–10 mm intervals and stained using hematoxylin and eosin. Horizontal sections were evaluated to identify potential skip lesions within the uterine corpus, defined as histologically confirmed lesions that were discontinuous from the primary cervical tumor and undetectable on preoperative imaging. Pathological assessments were conducted jointly by a board‐certified pathologist and a radiation oncologist. The definition of a skip lesion was established by consensus between both specialists. Exact binomial confidence intervals were calculated using the Clopper–Pearson method.

## 3 Results

The median age of the patients was 47.5 years (range: 32–74 years). Pathological evaluation revealed that 29 patients (58%) had FIGO Stage I disease (IA1, 2 cases; IB1, 18 cases; IB2, 7 cases; IB3, 2 cases), whereas 6 patients (12%) had FIGO Stage II disease (IIA1, 3 cases; IIB, 3 cases). Fifteen patients (30%) were classified as IIIC1. Patient and tumor characteristics are summarized in Table 1.

**TABLE 1:** Patient and tumor characteristics.

Number of patients		50
Age	Median (range)	47.5 (32–74)
Pathology	Squamous cell carcinoma	50 (100%)
Keratinization	Yes	13 (26%)
	No	37 (74%)
FIGO† Stage 2018	IA1	2 (4%)
	IB1	18 (36%)
	IB2	7 (14%)
	IB3	2 (4%)
	IIA1	3 (6%)
	IIB	3 (6%)
	IIIC1	15 (30%)
Surgical procedure	Simple hysterectomy	1 (2%)
	Radical hysterectomy	49 (98%)
Positive surgical margin	Yes	7 (14%)
	No	43 (86%)
Tumor size (mm)	Median (range)	32 (3–61)
Stromal invasion	> 1/3 of cervical stroma	40 (80%)
	< 1/3 of cervical stroma	10 (20%)
Lymphovascular space invasion	Yes	25 (50%)
	No	25 (50%)
Parametrial invasion	Yes	6 (12%)
	No	44 (88%)
Corpus invasion	Yes	4 (8%)
	No	46 (92%)
Regional lymph node metastases	Yes	15 (30%)
	No	35 (70%)
Common iliac node metastases	Yes	2 (4%)
	No	48 (96%)
Extranodal invasion	Yes	0 (0%)
	No	50 (100%)
Number of regional lymph node metastases	Median (range)	2 (1–5)

Abbreviation: FIGO^†^ = International Federation of Gynecology and Obstetrics.

Preoperative imaging studies, including MRI, did not identify any skip lesions in the uterine corpus. However, histopathological analysis revealed a CIS‐associated skip lesion in one case (2%; Clopper–Pearson 95% confidence intervals [CI]: 0.05%–10.5%). The patient with a CIS skip lesion had been clinically diagnosed preoperatively with FIGO Stage IA1 and subsequently underwent a simple hysterectomy. The primary tumor after biopsy measured 3 mm and was diagnosed as non‐keratinizing SCC without > 1/3 stromal invasion. No lymphovascular space invasion was observed, and there was no parametrial and corpus invasion. The CIS skip lesion was located in the endometrium of the uterine corpus, approximately 2 cm cranial to the primary tumor (Figure 1A), and measured approximately 2 mm in size (Figure 1C,B). This skip lesion was difficult to detect even on PET/CT scans.

**FIGURE 1:**
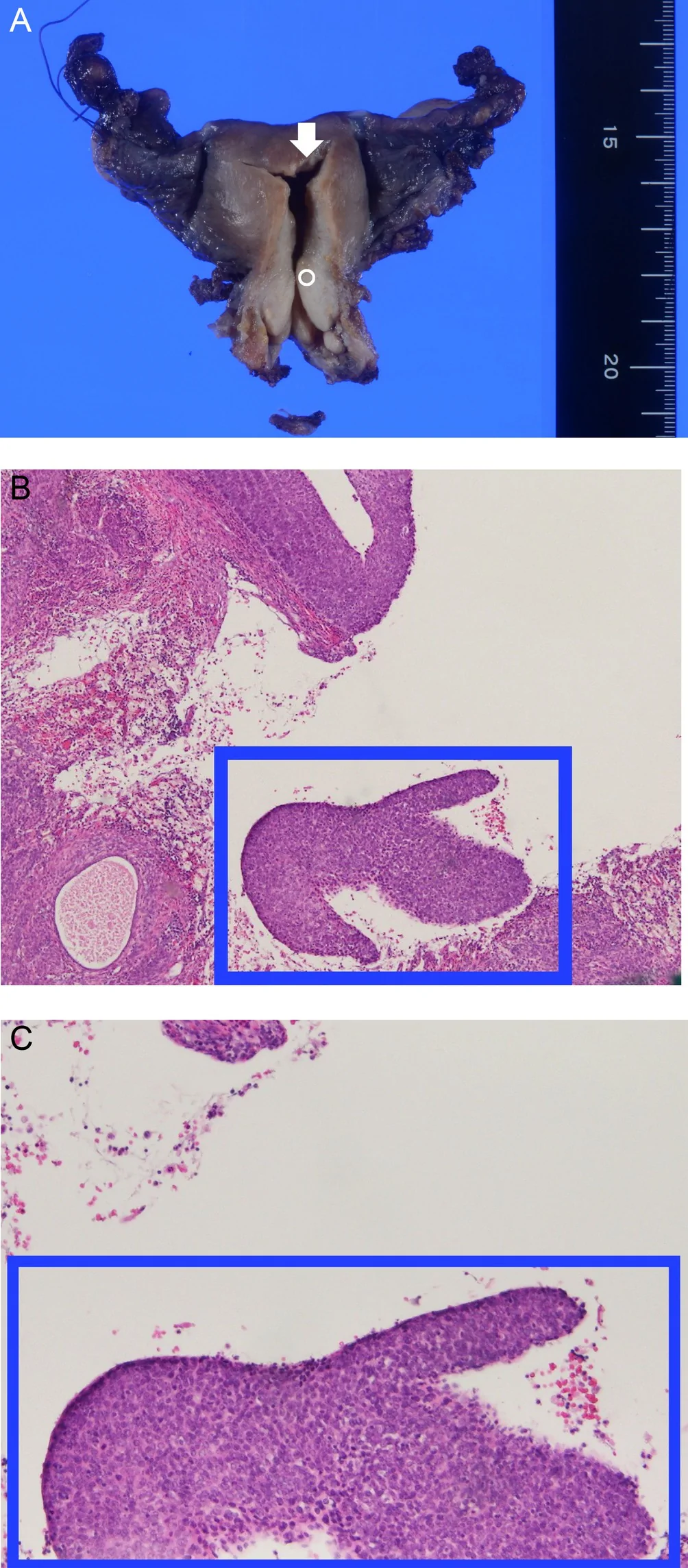
Carcinoma in situ (CIS) skip lesions in the uterine corpus are shown. (A) Macroscopic appearance of the uterine cervix. A CIS skip lesion (white arrow) located 2 cm cranial to the primary tumor after biopsy (white circle) is shown. (B) Hematoxylin and eosin‐stained histological sections illustrate a CIS skip lesion (blue boxes). (C) Magnified view of the CIS skip lesion.

## 4 Discussion

In our study cohort, CIS skip lesions in the uterine corpus were identified in 2% (Clopper–Pearson 95% CI: 0.05%–10.5%) of cases, demonstrating that, although rare, such lesions can occur. This finding suggests that, in radiotherapy for cervical SCC, potential extension of disease into the uterine corpus should be considered when determining treatment fields.

Damme et al. prospectively evaluated 21 patients with cervical cancer (16 SCCs and 5 adenocarcinomas) who received EBRT with concurrent chemotherapy, delivered with at least a 12 mm margin beyond the tumor, followed by hysterectomy [6]. The FIGO stages included Stage I (*n* = 2), Stage II (*n* = 14), Stage III (*n* = 3), and Stage IV (*n* = 2). They reported no residual tumor in the non‐targeted uterine corpus on pathological examination; however, the mean dose to the non‐targeted uterus was 41.22 Gy, indicating that a substantial incidental dose had been delivered.

Lee et al. retrospectively compared clinical outcomes between 128 patients treated with whole‐uterus irradiation (116 SCCs and 12 adenocarcinomas) and 92 patients treated with individualized uterine irradiation (81 SCCs and 11 adenocarcinomas) [7]. In the individualized approach, a margin of at least 15 mm beyond the tumor was applied. In the whole‐uterus irradiation group, brachytherapy was delivered using point A prescription in 75.8% and image‐guided brachytherapy in 24.2% of cases. In the individualized group, point A brachytherapy and image‐guided brachytherapy were used in 94.6% and 5.4% of cases, respectively. The 5‐year local control rates were 4.1% and 0% in the whole uterus and individualized irradiation groups, respectively, with no significant difference.

Hui et al. analyzed 109 patients treated with definitive chemoradiotherapy, including IMRT and brachytherapy [8]. They compared patients whose planning target volume encompassed ≥ 75% of the uterus with those in whom < 75% of the uterus was included. The cumulative incidence of local recurrence did not differ significantly between the two groups.

Collectively, these studies indicate that individualized uterine irradiation reduces the risk of treatment‐related toxicity without increasing the risk of local recurrence. This may suggest that incidental radiation dose is sufficient to control CIS lesions, or that CIS skip lesions in cervical SCC are rare and have limited impact on treatment outcomes. Nonetheless, when adopting individualized uterine irradiation, consideration should be given to the possibility of skip lesions.

This study has certain limitations. First, the sample size was small. Second, the cohort consisted of surgical cases; patients who are candidates for radiotherapy typically present with more advanced disease, and therefore the incidence of CIS skip lesions may be higher in a radiotherapy‐treated population. Third, the sampling resolution (5–10 mm intervals) may have underestimated small or localized CIS lesions.

In cervical SCC, CIS skip lesions in the uterine corpus were identified in 2% (Clopper–Pearson 95% CI: 0.05%–10.5%) of cases, confirming that although rare, they do occur. When determining radiotherapy target volumes, clinicians should recognize the potential presence of such lesions and ensure that at least a prophylactic dose is delivered to the uterine corpus.

## Author Contributions

**Mayumi Harada:** data curation, investigation, conceptualization, methodology, project administration, writing – original draft. **Yasuhito Hagiwara:** conceptualization, methodology, data curation, investigation, project administration, writing – original draft. **Takanobu Kabasawa:** data curation, investigation, methodology, writing – review and editing. **Hiraku Sato:** writing – review and editing. **Mayumi Ichikawa:** writing – review and editing. **Hiroko Akamatsu:** writing – review and editing. **Takashi Kaneko:** writing – review and editing. **Natsuko Yano:** writing – review and editing. **Ken Uematsu:** writing – review and editing. **Masanori Komatsu:** writing – review and editing. **Taiyo Tanae:** writing – review and editing. **Kenji Nemoto:** supervision, writing – review and editing. **Masashi Koto:** writing – review and editing, supervision.

## Funding

The authors have nothing to report.

## Conflicts of Interest

The authors declare no conflicts of interest.

## Data Availability

The data that support the findings of this study are available from the corresponding author upon reasonable request.
